# Relationship Between Short Video Addiction Tendency and Depression Among Rural Older Adults: Cross-Sectional Study

**DOI:** 10.2196/75938

**Published:** 2025-06-30

**Authors:** Ping Dong, Xianqi Zhang, Wenqiang Yin, Yongli Shi, Mengyuan Xu, Haoqi Li, Xianglan Zhuge, Ziyuan Li, Kui Sun, Zhongming Chen

**Affiliations:** 1 School of Management Shandong Second Medical University Weifang China; 2 School of Public Health Shandong Second Medical University Weifang China

**Keywords:** short video addiction, social media, mental health, depression, visual fatigue, sleep, rural community, older adults

## Abstract

**Background:**

Depression, a prevalent mental illness among older people, is associated with some adverse health problems and lower quality of life. Against the backdrop of a growing aging population, coping with late-life depression has become an important public health priority. Emerging evidence suggests that short video addiction tendency may be a new risk factor for depression. However, there has been limited discussion on the potential association between short video addiction tendency and depression among older adults.

**Objective:**

We aimed to investigate the relationship between short video addiction tendency and depression in a sample of rural older adults. In addition, we aimed to examine the mediating roles of asthenopia and sleep efficiency in the relationship between short video addiction tendency and depression.

**Methods:**

A face-to-face interview design was used to collect valid data from 872 rural older adults aged ≥60 years from October 2024 to January 2025 in 2 provinces of China. Participants were requested to complete self-report measures on short video addiction tendency (Short Video Addiction Scale), asthenopia (11-item Asthenopia Survey Questionnaire), sleep efficiency (actual sleep time and time in bed at night), and depression (Center for Epidemiologic Studies Depression Scale). Linear regression analyses were performed using model 6 of the PROCESS 4.1 macro in SPSS 26.0 to assess the relationship between short video addiction tendency and depression and to examine the mediating roles of asthenopia and sleep efficiency in this relationship, adjusting for sex, age, education, and marital status.

**Results:**

We observed that the prevalence of depression was 27.8% (242/872) in this study. There was a significant positive relationship between short video addiction tendency and depression (β=.263; *P*<.001). Short video addiction tendency affected depression through 3 different pathways: the mediating role of asthenopia (β=.084, 95% CI .059-.114); the mediating role of sleep efficiency (β=.021, 95% CI .001-.043); and the chain mediating role of asthenopia and sleep efficiency (β=.017, 95% CI .010-.026). The effect values of the 3 pathways accounted for 31.94%, 7.99%, and 6.46% of the total effect, respectively.

**Conclusions:**

We highlighted a direct and statistically substantial relationship between short video addiction tendency and depression, with asthenopia and sleep efficiency serving as potential mediating factors. Our findings predicted that guiding and assisting rural older adults to use short video apps appropriately, addressing asthenopia, and enhancing sleep efficiency may be a valuable approach to improve their mental health, preventing and delaying the occurrence and development of depression.

## Introduction

### Background

Since 2000, the situation of population aging in China has become increasingly serious, and it has now entered a moderately aging society, followed by a high degree of concern on health-related issues among older people. As one of the most common mental illnesses experienced by the older adults, depression, a mood disorder characterized by marked and persistent low mood and lack of fun, has become a major public health problem in China and even around the world [1]. It not only has a direct impact on the quality of life of older adults [2] but also increases the risk of frailty, dementia, and suicide [3-5]. According to the data from the World Health Organization [6], about 280 million people worldwide experienced different degrees of depressive symptoms. The proportion of patients with depression aged ≥60 years in the total population was 5.7%. A recent study [7] showed that the prevalence of depression among the rural older adults aged ≥60 years was as high as 47.43%, and the risk of depression was 1.38 times higher than that of the urban older adults [8], which meant that the older people living in rural areas faced greater mental health-related challenges. Late-life depression can reduce the quality of life and sense of well-being of older adults, as well as impose a heavy burden on their families and even society, thus affecting the realization of the goals of the healthy aging strategy. Therefore, early identification of the potential population at risk of depression and targeted interventions are crucial. A series of previous studies have revealed that depression is influenced by diverse factors, such as religious beliefs, self-rated health, residential status, level of food security, physical activity, and social isolation [9-11]. In addition, a recent study [12] has found that short video addiction may become a new risk factor for depression among older adults in the context of the increasing development of IT and the growing popularity of devices connected to the internet.

### Linking Short Video Addiction Tendency With Depression

As an emerging social media software, short video is increasingly favored by internet users for its concise and rich content. According to relevant statistics [13], the scale of short video users in China has reached 1.05 billion, accounting for 95.5% of the total number of internet users, and short video has become an important app to attract individuals to use the internet. In a recent survey with a sample of 2000 older adults [14], it was found that 75.1% had experience in short video creation and about 65% often watched >10 types of short videos. These results indicated that short video platforms have become an important channel for older adults to express themselves and share their lives, reflecting their high acceptance of and enthusiasm for participation in the rising internet culture. Previous studies have confirmed that using short video apps appropriately can help older adults reduce loneliness, enhance their connection to society, and improve self-efficacy and subjective well-being [15-18]. However, the possible negative effects of short video use also require considerable attention. Xiong et al [19] defined short video addiction tendency as symptoms of physiological and psychological discomfort caused by an individual’s irrational use of short videos. Short video apps, characterized by fragmented patterns, can show users what they may be interested in within a short period by intelligent algorithms [20], which could trigger addiction. Older adults have shorter exposure to the internet, making electronic devices and applications more tempting to them. Meanwhile, compared with older adults in urban areas, those in rural areas have lower levels of health literacy [21] and weaker self-discipline regarding maintaining a healthy behavioral lifestyle; thus, they become more susceptible to short video dependence or addiction. In addition, older people’s bodily functions remain in a state of constant weakness. Compared with other age groups, unreasonable short video use may expose older adults to more serious health threats. This issue warrants more attention and further research. Mu et al [22], in a study investigating the relationship between different types of web-based activities and depression among older adults, discovered that watching short videos can significantly aggravate depressive symptoms. The result of research conducted by Zhu et al [12] showed that there was a significant positive association between short video addiction and depression, with addicted users exhibiting higher levels of depression. Chao et al [23] also reached a consistent conclusion. Although experts have confirmed the relationship between short video addiction tendency and depression, the underlying mechanism of this relationship remains unclear. More research is needed to explore this further.

### Mediators: Asthenopia and Sleep Efficiency

Asthenopia refers to a group of symptoms involving visual impairment and ocular discomfort with or without systemic symptoms after long-term or high-intensity work, which exceeds the normal visual load of the eyes [24]. It includes a variety of symptoms such as eye dryness, eye soreness, blurred vision, and eye tearing [25], which can affect individuals’ learning, work, and life to different degrees [26-28]. In a study conducted in China in 2024 among 2870 adults aged 18 to 83 years [29], it was reported that the prevalence of asthenopia was 40.4%. It was also found that the prevalence of asthenopia increased with age by analyzing different age groups; in particular, participants aged ≥50 years had a prevalence of asthenopia as high as 54% [29]. Previous studies have shown that electronic device use can increase the risk of asthenopia. In a cross-sectional survey among Chinese university students [30], the risk of asthenopia was 2.2 times higher for students who relied on digital devices than for those who did not. A controlled study performed in Korea [31] indicated that smartphone use can worsen ocular symptoms, such as eye fatigue and dryness. Some scholars defined visual fatigue caused by viewing computer screens as computer vision syndrome [32]. In addition, many studies have been conducted to examine the relationship between asthenopia and depression. Han et al [33] discovered that asthenopia was significantly associated with negative mental status and a lower sense of social identity. A study in Saudi Arabia [34] revealed that problems on ocular surface health, including foreign body sensation, blurred vision, and pruritus, were closely associated with anxiety and depression. In other words, anxiety and depression may be more prevalent in groups with ocular surface discomfort. Therefore, we speculated that the effect of short video addiction tendency on depression may be mediated by asthenopia.

Sleep is a modifiable behavior and is highly intervenable [35]. Problematic sleep conditions, such as insomnia and obstructive sleep apnea, increase with age among older adults [36]. A meta-analysis [37] suggested that the proportion of individuals with sleep disorders in older adults was 30.5%, indicating that more attention should be paid to the sleep health of this group. Sleep efficiency has been recognized as an objective indicator of sleep quality, acquired by measuring time in bed and actual sleep time [38]. Extensive evidence has shown that low sleep efficiency is one of the prominent problems that threaten the physical and mental health, as well as the quality of life, of older adults [39-41]. In general, the optimal length of sleep for older people is between 7 and 8 hours [42]. However, a recent study [43] found that 30.67% of older adults slept <7 hours and 33.65% slept >8 hours. Only 41.59% had a sleep efficiency >85%. The proportion of older adults whose sleep efficiency was <65% reached 24.29% [44]. Christensen et al [45] observed that sleep efficiency can be influenced by screen use. Specifically, individuals with longer screen use tended to have lower sleep efficiency. A Turkish study involving high-school students [46] reported that students with addictive psychology or behaviors toward social media were more likely to have low sleep efficiency. At the same time, several studies have demonstrated that sleep efficiency can significantly predict depression [47,48]. Yan et al [49] found that a low level of sleep efficiency can markedly increase the risk of depression. In addition, they found that compared with women, sleep efficiency was more closely associated with the incidence of depression in men. Researchers also verified a positive association between variability in sleep efficiency and severity of anxiety and depression [50]. Furthermore, previous evidence has confirmed that asthenopia is associated with sleep efficiency [51]. A study based on data from the Korean National Health and Nutrition Examination Survey [52] showed that eye irritation symptoms can cause difficulty sleeping and shorten sleep time. A survey conducted in China among ophthalmologists [53] found that patients with asthenopia may experience poorer sleep quality and shorter sleep duration than individuals without asthenopia. Therefore, we hypothesized that sleep efficiency was a mediator between short video addiction tendency and depression, and that asthenopia and sleep efficiency played a chain mediating role between short video addiction tendency and depression.

### This Study

While some studies have confirmed that asthenopia and sleep efficiency are related to short video addiction tendency and depression [30,49], it is not yet clear how they affect this relationship. As far as we know, this is the first study to consider the mediating role of asthenopia and sleep efficiency. Moreover, until now, studies specifically focusing on short video addiction and asthenopia among older adults have remained limited. Thus, this study examined the mediating roles of asthenopia and sleep efficiency in the relationship between short video addiction tendency and depression using a sample of rural older adults. On the basis of previous empirical research, we proposed four hypotheses: (1) short video addiction tendency can positively predict depression; (2) short video addiction tendency can indirectly predict depression through the mediating effect of asthenopia; (3) short video addiction tendency can indirectly predict depression through the mediating effect of sleep efficiency; (4) short video addiction tendency can indirectly predict depression through the chain mediation of asthenopia and sleep efficiency.

## Methods

### Participants

This study was conducted from October 2024 to January 2025 using a multistage stratified sampling method. First, 2 provinces were extracted as sample areas according to the high and low levels of economic development. Then, 1 prefecture-level city was chosen from each sample province, from which 3 districts (counties) were selected according to high, medium, and low levels of economic development. Because the difference between the total populations of the sample districts (counties) in the 2 provinces were relatively small, we adopted an equal-weight sampling method. In total, 4 townships (streets) were chosen from each district (county), and 6 villages were selected from each township. We randomly selected 23 older adults (3 of them were incremental samples calculated according to a 15% loss to follow-up rate) from each village for this survey. Recruitment quotas were distributed with reference to the age structure of older people in each village. Trained investigators conducted paper format surveys in a face-to-face manner on the spot. Each questionnaire took nearly 20 minutes. A total of 3216 responses were returned, and the valid response rate was 98.7% (3175/3216), excluding invalid responses (incomplete and logically incorrect answers). Finally, according to the objective of this study, 872 short video users aged ≥60 years residing in rural areas were eligible to be chosen as the participants.

### Ethical Considerations

The study was approved by the Medical Ethics Committee of Weifang Medical University (2021YX-066) and performed in accordance with the ethical guidelines of the Declaration of Helsinki. Written informed consent was obtained from each participant before the survey. All data were fully anonymized and securely stored. The participants did not receive any remuneration.

### Measures

#### Depression

The Center for Epidemiologic Studies Depression Scale was used to assess the frequency of depressive symptoms in the past week [54]. The scale consists of 10 items on a 4-point Likert scale ranging from “0=rarely or not at all” to “3=most of the time.” The scores of all items were added together to obtain a total score, which ranges from 0 to 30. The higher the score, the more severe the depression. A total score of >10 suggests that the individual may be depressed [55]. Center for Epidemiologic Studies Depression Scale demonstrated excellent psychometric properties in the Chinese population [56]. In this study, its Cronbach α coefficient was 0.887, indicating good reliability. The Kaiser-Meyer-Olkin (KMO) value was 0.915, and the Bartlett sphericity test yielded *χ^2^*_45_=4038.9 (*P*<.001), indicating that it was suitable for factor analysis. Through exploratory factor analysis, we extracted 2 common factors, with a cumulative variance contribution rate of 61.125%. Each item loaded >0.5 on a certain common factor, indicating good structural validity.

#### Short Video Addiction Tendency

We used the Short Video Addiction Scale, which was adapted by Zhang et al [57] based on the Social Network Services Addiction Scale [58], to evaluate the status of individual short video addiction tendency. The scale is composed of 6 items on a 7-point Likert scale. The range of total scores is 6 to 42. Higher scores indicate higher reliance on short video apps, which implies higher levels of short video addiction tendency. The reliability and validity of the Short Video Addiction Scale have been verified in China [57]. In our sample, it yielded a Cronbach α of 0.911, indicating good reliability. The KMO value was 0.886, and the Bartlett sphericity test yielded *χ^2^*_15_=3394.4 (*P*<.001), indicating that it was suitable for factor analysis. The factor analysis suggested that there was a unidimensional factor structure, and the cumulative variance contribution rate was 69.479%. Each item loaded >0.8 on a certain common factor, indicating good structural validity.

#### Asthenopia

The asthenopia was measured by the 11-item Asthenopia Survey Questionnaire. The scale contains 11 items divided into 2 dimensions: symptoms concerning the eye and symptoms concerning visual function and the whole body. Each item was rated from 0 to 4 (0=none, 1=mild, 2=moderate, 3=relatively severe, and 4=extremely severe). Cumulative scores range from 0 to 44. The higher the score, the more severe the symptoms of asthenopia are. Those who had scores of >8 were classified as having asthenopia. The 11-item Asthenopia Survey Questionnaire has been proven to be valid and reliable for the Chinese population [59]. The Cronbach α coefficient was 0.913 in this study, indicating good reliability. The KMO value was 0.902, and the Bartlett sphericity test yielded *χ^2^*_55_=5912.4 (*P*<.001), indicating that it was suitable for factor analysis. Through exploratory factor analysis, we extracted 2 common factors that contributed 65.541% cumulatively to the total variance. Each item loaded >0.5 on a certain common factor, indicating good structural validity.

#### Sleep Efficiency

Sleep efficiency is the ratio of actual sleep time to the time in bed at night [38]. In particular, time in bed = sleep onset latency + total sleep time + time awake after sleep onset but before final awakening + time attempting to sleep after the final awakening [60]. Sleep efficiency in this study was categorized according to the Pittsburgh Sleep Quality Index Scale: 0=<65%, 1=65%-74%, 2=75%-84%, and 3=≥85%. Higher scores indicate higher sleep efficiency.

#### Covariates

The covariates included sex (male or female); age (60-64 years, 65-69 years, 70-74 years, and ≥75 years); education (primary school or lower, middle school, and high school or higher); and marital status (single or married).

### Statistical Analysis

Descriptive analysis was used to present the basic demographic characteristics of the participants and the scores of key variables. We applied the Pearson correlation analysis to examine the association between the key variables. The PROCESS macro has become the preferred tool for many disciplines because of its efficiency and rigor, especially for models in which all variables are explicit [61]. Thus, adjusting for sex, age, education, and marital status, we used the model 6 of the PROCESS 4.1 macro [62] for mediation analysis: (1) linear regression analysis to explore the relationship between short video addiction tendency and depression; (2) linear regression analysis to test the relationship between short video addiction tendency and asthenopia; (3) linear regression analysis to test the associations of short video addiction tendency and asthenopia with sleep efficiency; and (4) linear regression analysis to further explore the relationship between short video addiction tendency and depression, with asthenopia and sleep efficiency as mediators. Finally, the mediating effect was further verified based on the bootstrap method. If 0 was not included in the 95% CI, the results could be considered statistically significant. In this study, the data processing and analyses were performed using SPSS 26.0. *P*<.05 (2-tailed) was set as the significance level.

## Results

### Sample Characteristics

Table 1 shows that among the 872 participants, 356 (40.8%) were male and 516 (59.2%) were female. Most (718/872, 82.3%) of them were aged between 60 and 74 years. Nearly half (431/872, 49.4%) of the participants had the highest education level of primary school or lower. In total, 80.8% (705/872) of the participants were married. The mean score for short video addiction tendency was 12.12 (SD 7.56). The mean score for asthenopia was 6.72 (SD 6.79). A total of 34.9% (304/872) of the participants were reported to have asthenopia. The mean scores for sleep efficiency and depression were 1.82 (SD 1.16) and 7.52 (SD 6.08), respectively. The prevalence of depression was 27.8% (242/872).

**Table 1:** Characteristics of the participants (N=872).

Variables		Values
**Sex** **, n (%)**		
	Male	356 (40.8)
	Female	516 (59.2)
**Age (y), n (%)**		
	60-64	252 (28.9)
	65-69	225 (25.8)
	70-74	241 (27.6)
	≥75	154 (17.7)
**Education, n (%)**		
	Primary school or lower	431 (49.4)
	Middle school	320 (36.7)
	High school or higher	121 (13.9)
**Marital status, n (%)**		
	Single^a^	167 (19.2)
	Married	705 (80.8)
Short video addiction tendency, mean (SD)		12.12 (7.56)
Asthenopia, mean (SD)		6.72 (6.79)
Sleep efficiency, mean (SD)		1.82 (1.16)
Depression, mean (SD)		7.52 (6.08)

^a^Single includes single, divorced, and widowed.

### Correlation Between Key Variables

Table 2 presents the correlation between short video addiction tendency, asthenopia, sleep efficiency, and depression. We found that short video addiction tendency was positively correlated with asthenopia (*r*=0.301; *P*<.001) and depression (*r*=0.266; *P*<.001) and negatively correlated with sleep efficiency (*r*=−0.129; *P*<.001). There was a negative correlation between asthenopia and sleep efficiency (*r*=−0.228; *P*<.001), and a positive correlation between asthenopia and depression (*r*=0.390; *P*<.001). Sleep efficiency was negatively associated with depression (*r*=−0.380; *P*<.001). In accordance with the conventional guidelines for interpretation [63], all correlations between the key variables were weak (range 0.10-0.39).

**Table 2:** Correlation between short video addiction tendency, asthenopia, sleep efficiency, and depression.

Variables			Short video addiction tendency		Asthenopia		Sleep efficiency		Depression
**Short video addiction tendency**									
	*r*	1		0.301		−0.129		0.266	
	*P* value	—^a^		<.001		<.001		<.001	
**Asthenopia**									
	*r*	0.301		1		−0.228		0.390	
	*P* value	<.001		—		<.001		<.001	
**Sleep efficiency**									
	*r*	−0.129		−0.228		1		−0.380	
	*P* value	<.001		<.001		—		<.001	
**Depression**									
	*r*	0.266		0.390		−0.380		1	
	*P* value	<.001		<.001		<.001		—	

^a^Not applicable.

### Mediation Effect Analysis

**Figure 1:**
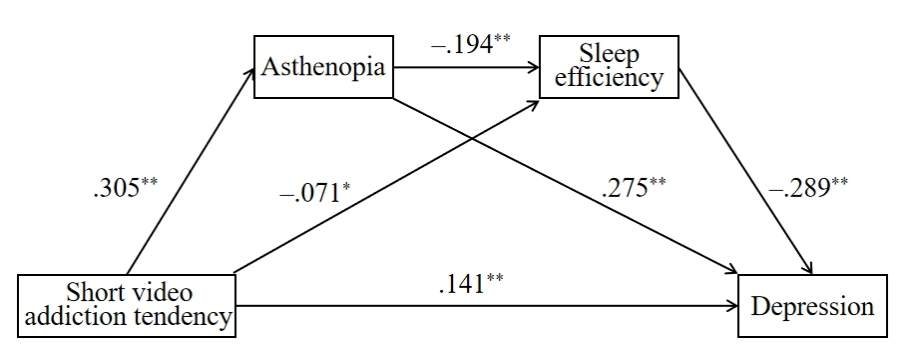
Path diagram of the impact of short video addiction tendency on depression. The models control for sex, age, education, and marital status. **P<.001; *P=.04.

As shown in Table 3, by controlling for sex, age, education, and marital status, short video addiction tendency positively predicted asthenopia (β=.305; *P*<.001) and depression (β=.141; *P*<.001) and negatively predicted sleep efficiency (β=−.071; *P*=.04). Rural older people with high short video addiction tendency had a higher risk of asthenopia and depression as well as lower sleep efficiency. Asthenopia positively predicted depression (β=.275; *P*<.001) and negatively predicted sleep efficiency (β=−.194; *P*<.001). Rural older adults with asthenopia had a higher risk of depression and lower sleep efficiency. In addition, sleep efficiency negatively predicted depression (β=−.289; *P*<.001), which suggested that their depressive symptoms worsened as sleep efficiency decreased. Therefore, the short video addiction tendency can indirectly predict depression through the chain mediation of asthenopia and sleep efficiency. Figure 1 shows the chain mediation model.

**Table 3:** Analysis of the mediation effect of short video addiction tendency on depression.

Outcome variables and predictors		*R*	*R* ^2^	*F* test (*df*)	*P* value	β	*t* test (*df*)	*P* value
**Asthenopia**		0.320	0.102	19.691 (5, 866)	<.001			
	Short video addiction tendency					.305	9.430 (866)	<.001
	Sex					.084	2.501 (866)	.01
	Age					−.020	−0.590 (866)	.56
	Education					−.044	−1.274 (866)	.20
	Marital status					−.021	−0.640 (866)	.52
**Sleep efficiency**		0.271	0.073	11.417 (6, 865)	<.001			
	Short video addiction tendency					−.071	−2.056 (865)	.04
	Asthenopia					−.194	−5.622 (865)	<.001
	Sex					−.063	−1.841 (865)	.07
	Age					.002	0.051 (865)	.96
	Education					.104	2.979 (865)	.003
	Marital status					.002	0.048 (865)	.96
**Depression**		0.519	0.269	45.381 (7, 864)	<.001			
	Short video addiction tendency					.141	4.579 (864)	<.001
	Asthenopia					.275	8.808 (864)	<.001
	Sleep efficiency					−.289	−9.568 (864)	<.001
	Sex					−.010	−0.323 (864)	.75
	Age					−.020	−0.646 (864)	.52
	Education					−.069	−2.206 (864)	.03
	Marital status					−.066	−2.205 (864)	.03
**Depression**		0.305	0.093	17.699 (5, 866)	<.001			
	Short video addiction tendency					.263	8.073 (866)	<.001
	Sex					.036	1.073 (866)	.28
	Age					−.027	−0.790 (866)	.43
	Education					−.114	−3.287 (866)	.001
	Marital status					−.073	−2.207 (866)	.03

Table 4 displays the results of the mediating effects test based on the bias-corrected bootstrapping procedure. The direct effect of short video addiction tendency on depression was .141 (95% CI .065-.162), accounting for 53.61% of the total effect. The total indirect effect was .122 (95% CI .088-.159), accounting for 46.39% of the total effect, and it consisted of 3 paths: path 1 was short video addiction tendency→asthenopia→depression (β=.084, 95% CI .059-.114), path 2 was short video addiction→sleep efficiency→depression (β=.021, 95% CI .001-.043), and path 3 was short video addiction→asthenopia→sleep efficiency→depression (β=.017, 95% CI .010-.026). The ratios of the indirect effects of paths 1, 2, and 3 to the total effect were 31.94%, 7.99%, and 6.46%, respectively. By comparing all effect values, we found that the direct effect of short video addiction tendency on depression was dominant, followed by the indirect path 1.

**Table 4:** Testing the pathways of the mediation model.

Pathway	β (SE; 95% CI)	Mediation (%)
Total effect	.263 (.026; .160-.263)	100
Direct effects	.141 (.025; .065-.162)	53.61
Total indirect effect	.122 (.018; .088-.159)	46.39
Indirect path 1	.084 (.014; .059-.114)	31.94
Indirect path 2	.021 (.011; .001-.043)	7.99
Indirect path 3	.017 (.004; .010-.026)	6.46

## Discussion

### Principal Findings

Depression is a challenging problem in geriatric health management. This study is the first to examine the mediating role of asthenopia and sleep efficiency in the relationship between short video addiction tendency and depression among rural older adults. Our findings were complementary to existing studies, which deepened the understanding of the mechanism between short video addiction tendency and depression and provided new ideas and evidence for effective prevention and intervention strategies for depression among older adults in the future.

We verified the relationship between short video addiction tendency and depression. This study found that the short video addiction tendency of rural older adults had a significant positive predictive effect on the risk of depression, which was consistent with previous studies [64,65]. The reason for this may be that users of social media platforms, such as short video apps, are more accustomed to uploading pleasant information about their successes and experiences, which makes other users unconsciously fall into the trap of making upward comparisons [66]. After realizing the gap between themselves and others, they may develop pessimistic emotions such as frustration and anxiety, reducing their life satisfaction and sense of well-being, which can have a negative impact on their mental health and even lead to depression [67]. Besides, short video apps can recommend personalized content that users may be interested in through intelligent algorithms, which can form information cocoons that lack diversity [68]. It can affect an individual’s mental health from 2 aspects: on the one hand, the platform may recommend more content that tends to trigger upward comparisons to the account, thereby reinforcing the impact of this psychological behavior; on the other hand, the information cocoon caused by algorithms can inhibit an individual’s exposure to new things, resulting in a restricted horizon. If individuals watch short videos with highly similar content for a long time, their sense of satisfaction and happiness will continue to weaken, which will lead to boredom, irritability, and other negative emotions. Previous research has revealed that addiction to short video apps occupies time and opportunities for social interaction in real life and intensifies the indifference to real-life relationships, which ultimately makes it difficult for individuals to obtain timely and adequate emotional needs and social support and cause varying degrees of social maladjustment, increasing their loneliness and depressive symptoms [69]. In addition, it has been found that prolonged screen use and depression could potentially be linked due to pain in certain parts of the body, such as the head and neck [70]. Meanwhile, older people have lower levels of digital literacy [71]. Weak cybersecurity awareness and poor ability to recognize false and fraudulent information can increase the probability of exposure to negative cyber events [72]. They may also develop cognitive biases due to misleading content, such as inappropriate interpretations of health information, leading to more intense feelings of helplessness and depressive symptoms. In addition, the established evidence [73] suggests that people with low digital literacy have more difficulty in recognizing the presence of addiction and taking appropriate actions in time to adjust, thus exacerbating the detrimental effects of short video addiction on mental and physical health.

In this study, we confirmed the mediating role of asthenopia in the relationship between short video addiction tendency and depression for the first time. We found that if rural older adults had high levels of dependence on short video apps, they may experience asthenopia, thereby increasing the risk of depression. First, a short video addiction tendency can increase the risk of asthenopia. Mobile phones can emit more radiation with increasing frequency and duration of use, leading to oxidative stress in the corneal and lens tissues [74]; thus, exacerbating symptoms of ocular discomfort, such as eye pain, dry eyes, and photophobia. Previous studies have suggested [75,76] that screen time is associated with blink frequency and eyelid closure, which can affect ocular comfort and visual clarity by decreasing tear film stability and increasing pressure on the cornea. Moreover, watching short videos implies that an individual’s eyes are continuously in focus for a certain period [77], increasing the pressure on eye movements, which is, in essence, an important mechanism to contribute to asthenopia. Second, previous research [78,79] has shown that issues related to ocular surface health are risk factors for psychiatric systemic disorders. Ocular discomfort symptoms can significantly increase an individual’s mental stress and reduce subjective well-being and quality of life [80,81], resulting in an increased risk of depression. Meher and Gharge [82] concluded that visual impairment prevented individuals from effectively communicating and interacting with others, making them more likely to feel lonely and socially isolated, and more prone to depression. Furthermore, asthenopia may negatively impact an individual’s mental health through physical functional limitations [83]. A recent study [84] has proven that there was a significant positive dose-response relationship between the levels of physical activity and mental health status. Appropriate physical activity plays a crucial role in enhancing life satisfaction and prevention and intervention for depression [85].

Our study also demonstrated the mediating role of sleep efficiency in the relationship between short video addiction tendency and depression. This implies that short video addiction tendency may decrease an individual’s sleep efficiency, which in turn leads to the occurrence of depression. First, short video addiction tendency was associated with a subsequent decline in sleep efficiency, which is in line with published evidence [45]. Watching short videos before going to bed can lead to conscious or unconscious sleep delay behavior [86], shortening the duration of sleep. Immersion theory [87] is considered able to appropriately explain this point, that is, when individuals are immersed in short video apps, they tend to reduce their perception of time. Other studies have shown that nighttime screen exposure inhibits the pineal gland from synthesizing and secreting melatonin [88], which is a marker that regulates circadian rhythms [89]. Decreased levels of melatonin can trigger a range of sleep disorders, such as difficulty in falling asleep and an increased number of nighttime awakenings [90]. In addition, according to the sleep disruption process theory [91,92], watching short videos before bedtime may stimulate neuronal cells in the brain through cognitive and emotional arousal, making it difficult to create favorable sleep preparation conditions. Second, sleep efficiency can positively predict depression. Jaussent et al [93] suggested that difficulty in falling asleep and maintaining sleep were independent risk factors for depressive symptoms. Lack of sleep can lead to uncontrolled emotional regulation, which destroys the stability of emotions, and may accelerate the occurrence and development of depression in severe cases [94]. Cognitive impairment may also be a bridge between sleep efficiency and depression. A meta- analysis [95] indicated that individuals with mild cognitive impairment had significantly lower sleep efficiency than those with healthy cognition, and poor sleep efficiency seemed to be relevant to subsequent cognitive impairment. It has been proven that sleep disorders impede the generation of cortical gaps and reduce the rate of clearing Aβ substances from the brain, which in turn leads to cognitive decline [96]. Meanwhile, people with cognitive impairment may be more susceptible to mood disorders and emotional apathy, which can promote depression [97,98]. Moreover, researchers found that neuroendocrine system disorders caused by poor sleep quality were prone to elevated levels of glucocorticoid hormones, melatonin dysregulation, and chronic inflammation, all of which were associated with the incidence of depression [99].

We also found a significant pathway of short video addiction tendency→asthenopia→sleep efficiency→depression in this study. The model indicates that the chain relationship between asthenopia and sleep efficiency also mediates the association between short video addiction tendency and depression. Numerous studies [100,101] have shown that the higher the degree of short video addiction tendency, the more pronounced the symptoms of asthenopia. There is a significant association between asthenopia and reduced sleep efficiency [83,102], and low sleep efficiency is a risk factor for depression [103]. In other words, the higher the level of short video addiction tendency exhibited by rural older adults, the more severe asthenopia they may have. Furthermore, these adverse symptoms associated with asthenopia can further reduce sleep efficiency, thereby threatening their mental health. This model tells us that poorer sleep efficiency is probably the result of asthenopia. When rural older people experience asthenopia, they are more likely to have adverse sleep problems, which can increase the risk of depression.

### Limitations

Certain limitations should be recognized in this study. First, the causality between the study variables could not be ascertained due to the design of the cross-sectional survey. Second, the data used in the study were obtained from self-report procedures, which may be subject to information bias. Third, except for asthenopia and sleep efficiency, there may be other mediating variables in the relationship between short video addiction tendency and depression, which need to be further explored in the future. Fourth, the covariates included in our study were limited, and some factors that may be related to the key variables were not observed. Finally, this study was restricted to rural areas of 2 provinces in China. To ensure the generalizability of the conclusions, follow-up studies should continue to expand the coverage of the survey in terms of population and region.

### Conclusions

This study extended the previous literature by providing insight into how short video addiction tendency contributes to depression among rural older adults. Our results indicated that a higher level of short video addiction tendency was associated with a higher risk of depression. Asthenopia and sleep efficiency can separately mediate the relationship between short video addiction tendency and depression. In addition, asthenopia and sleep efficiency could also sequentially mediate the relationship between short video addiction tendency and depression. Therefore, prevention and intervention programs for depression among rural older adults could be targeted to reduce the degree of short video addiction tendency, alleviate asthenopia, and enhance sleep efficiency.

## Abbreviations

KMO: Kaiser-Meyer-Olkin

## Data Availability

The datasets used and analyzed during this study are available from the corresponding authors upon reasonable request.
